# In Vitro Evaluation of the Effect of Microabrasion and Resin Infiltration Materials on Enamel Microhardness and Penetration Depth

**DOI:** 10.3390/jfb17020067

**Published:** 2026-01-29

**Authors:** Elif Ercan Devrimci, İdil Gönüllü, Hande Kemaloğlu, Murat Türkün, Ayşegül Demirbaş

**Affiliations:** Department of Restorative, Ege University, İzmir 35100, Türkiye; ercan.elif@ege.edu.tr (E.E.D.); hande.kemaloglu@ege.edu.tr (H.K.); murat.turkun@ege.edu.tr (M.T.); aysegul.demirbas@ege.edu.tr (A.D.)

**Keywords:** enamel microhardness, resin infiltration, microabrasion, demineralized enamel, penetration depth

## Abstract

**Background:** This in vitro study aimed to evaluate the effect of microabrasion as a surface pretreatment and to compare an experimental resin infiltrant with a commercially available system (ICON) in terms of enamel surface microhardness recovery and resin penetration depth in artificially demineralized enamel lesions. **Methods:** Forty-eight caries-free human third molars were prepared to obtain standardized enamel specimens, and artificial enamel lesions were created using a pH-cycling model. Specimens were randomly allocated into four groups (*n* = 12): experimental resin with microabrasion, experimental resin without microabrasion, ICON resin with microabrasion, and ICON resin without microabrasion. When indicated, microabrasion was performed using a 6.6% hydrochloric acid paste for a total application time of 30 s, followed by standard hydrochloric acid etching as part of the infiltration protocol. Enamel surface microhardness was measured at baseline, after demineralization, and after resin infiltration. Resin penetration depth was assessed using confocal laser scanning microscopy, with six specimens per group (*n* = 6). Data were analyzed using repeated-measures mixed-effects models and one-way ANOVA (*p* < 0.05). **Results:** Resin infiltration resulted in a partial recovery of enamel surface microhardness following demineralization; however, baseline hardness values were not fully restored, and no statistically significant differences were observed among the study groups (*p* > 0.05). These findings indicate surface stabilization rather than complete mechanical or mineral restoration. The ICON resin demonstrated significantly greater penetration depth than the experimental resin. In both resin systems, microabrasion significantly increased penetration depth. **Conclusions:** Within the limitations of this in vitro study, resin infiltration primarily contributed to the stabilization of demineralized enamel surfaces rather than true remineralization or full mechanical recovery. Although microabrasion enhanced resin penetration depth, this effect should be interpreted with caution due to the potential for cumulative enamel loss. From a clinical perspective, these findings support the selective use of microabrasion to enhance resin infiltration in early enamel lesions with pronounced surface barriers, while emphasizing the need to balance penetration benefits against enamel preservation.

## 1. Introduction

Dental caries is a globally prevalent, biofilm-mediated disease that causes progressive mineral loss of dental hard tissues, affects nearly 2.3 billion individuals worldwide, and constitutes the most common health condition with a significant economic burden [1,2].

In recent decades, the prevalence of cavitated caries lesions has declined due to improvements in preventive care, fluoride exposure, and advances in early diagnostic technologies. Nevertheless, enamel demineralization, commonly referred to as white spot lesions (WSLs), continues to be frequently observed, particularly in individuals exposed to prolonged acidic challenges or undergoing orthodontic treatment [3]. White spot lesions represent the earliest clinically detectable stage of dental caries, characterized by subsurface mineral loss while the surface enamel layer remains relatively intact [4]. Acidic byproducts produced by cariogenic bacteria play a central role in enamel demineralization, and WSLs have been reported in up to 96% of patients receiving fixed orthodontic appliances [5].

Treatment approaches for white spot lesions range from non-invasive to minimally invasive strategies, including topical fluoride therapy, casein phosphopeptide–amorphous calcium phosphate (CPP–ACP) applications, microabrasion, and restorative interventions. When detected at an early stage prior to cavitation, WSLs may undergo partial or complete regression through remineralization processes, and invasive restorative treatment is generally not recommended [6]. However, conventional remineralization techniques present several limitations, including restricted mineral deposition primarily at the lesion surface, prolonged treatment duration, and reduced effectiveness once lesions have progressed beyond the initial stage [6].

Resin infiltration has emerged as a minimally invasive treatment alternative for the management of non-cavitated enamel lesions. This technique is based on the penetration of a low-viscosity, light-polymerized resin into the porous structure of demineralized enamel. By occluding the microporosities within the lesion body, the infiltrant replaces lost mineral content, stabilizes the enamel structure, and effectively blocks diffusion pathways for acids, thereby arresting further lesion progression [7,8,9]. The penetration capacity of resin infiltrants is strongly influenced by their physicochemical properties, including low viscosity, high surface tension, and favorable contact angles with etched enamel surfaces [8,10,11,12]. Consistent with current evidence, including the Cochrane systematic review on micro-invasive interventions, resin infiltration has been shown to arrest the progression of non-cavitated caries lesions; however, variability in materials and treatment protocols highlights the need for further material-focused investigations [13].

Resin infiltration is primarily indicated for non-cavitated enamel lesions with an intact surface layer, such as early white spot lesions, whereas it is contraindicated in cavitated lesions, dentin involvement, or cases with extensive enamel loss where conventional restorative treatment is required.

Resin infiltration is limited to enamel lesions and is not indicated for demineralization extending into dentin, as resin penetration depth is insufficient to effectively infiltrate dentinal tissue.

The resin infiltration procedure consists of three essential steps: (1) surface conditioning, typically performed using 15% hydrochloric acid to remove the hypermineralized surface layer; (2) dehydration with ethanol to eliminate residual moisture within the lesion pores; and (3) application of a methacrylate-based infiltrant resin, predominantly composed of triethylene glycol dimethacrylate (TEGDMA) [8]. The commercial resin infiltration system ICON (DMG, Hamburg, Germany), introduced in 2009 through collaborative research between Charité University of Berlin and Kiel University, remains the most widely studied and clinically available infiltrant system [14].

The experimental resin infiltrant used in this study was a TEGDMA-based low-viscosity formulation designed to allow penetration into demineralized enamel microporosities.

Surface microhardness measurement is a well-established, non-destructive method for assessing mineral loss and recovery in enamel and dentin. This technique allows for sensitive detection of changes in mineral content by evaluating the resistance of the enamel surface to indentation forces, commonly expressed as Vickers or Knoop hardness values [15,16]. Consequently, microhardness testing is frequently employed in in vitro and in situ studies investigating demineralization, remineralization, and the effects of minimally invasive treatment modalities.

In addition to mechanical assessment, the evaluation of resin penetration depth within enamel lesions provides critical insight into the effectiveness of resin infiltration therapy. Confocal laser scanning microscopy (CLSM), combined with dual-fluorescence staining techniques, enables clear differentiation between infiltrated and non-infiltrated regions within the lesion body. Following resin infiltration, infiltrated areas become impermeable to secondary dyes, whereas non-infiltrated porous zones remain stained, allowing for precise visualization and semi-quantitative assessment of penetration depth [14,17].

Although resin infiltration has been demonstrated to be an effective method for arresting early caries lesions, the increasing clinical demand for infiltrant agents and the reliance on a single commercial product raise concerns regarding accessibility and cost. Consequently, the development and evaluation of experimental resin infiltration materials represent an important area of research aimed at expanding treatment options and improving clinical feasibility.

Therefore, the aim of the present in vitro study was to evaluate the effects of microabrasion as a surface pretreatment and to compare an experimental resin infiltrant with a commercially available ICON system in terms of enamel microhardness recovery and resin penetration depth in artificially demineralized enamel lesions. The following hypotheses were tested:

**H1:**  *The experimental resin infiltration agent increases the surface microhardness of demineralized enamel.*

**H2:**  *The experimental resin infiltration agent penetrates sufficiently into the body of enamel lesions.*

**H3:**  *Resin infiltration penetration depth varies depending on the inclusion of microabrasion as a surface pretreatment.*

## 2. Materials and Methods

### 2.1. Preparation of Enamel Samples

For the microhardness evaluation, 36 extracted human third molars with closed apices and free of caries were used following approval from the Ege University Faculty of Medicine Medical Research Ethics Committee (approval no. 25-2.1T/75). The extracted teeth were obtained for reasons unrelated to this study, including orthodontic and surgical indications. Teeth with caries, restorations, cracks, developmental defects, or visible enamel irregularities were excluded, and only intact, caries-free teeth with fully formed apices were included. Until the experimental procedures, the teeth were stored in a 0.1% thymol solution to prevent microbial growth.

The crowns were separated from the roots at the cemento–enamel junction and sectioned in the mesiodistal direction using a precision cutting device (Isomet 1000; Buehler, IL, USA) to obtain two enamel specimens from each tooth. Each specimen was embedded in acrylic resin blocks, leaving the enamel surface exposed. Each enamel specimen was prepared as a standardized block measuring approximately 4 × 4 × 2 mm (width × length × thickness). To standardize the surfaces and remove irregularities, enamel surfaces were sequentially polished using 180-, 400-, 600-, 1000-, and 2000-grit silicon carbide papers under running water for 15 s per grit (Mecapol P230, Presi, Grenoble, France).

### 2.2. Sample Allocation

The enamel specimens were randomly assigned to four experimental groups using a computer-generated randomization sequence created with SPSS software (v27.0, IBM Corp., Chicago, IL, USA). Specimens were allocated according to the resin infiltration system and the surface pretreatment protocol applied:

G1: Experimental resin infiltrant with prior microabrasion

G2: Experimental resin infiltrant without prior microabrasion

G3: ICON resin infiltrant with prior microabrasion

G4: ICON resin infiltrant without prior microabrasion

Sample size calculation was performed using power analysis with a 95% confidence level and 80% statistical power, resulting in a minimum of 12 specimens per group (*n* = 12).

### 2.3. Initial Surface Microhardness Measurement

Surface microhardness was measured using a Vickers hardness tester (Shimadzu HMV-2, Tokyo, Japan) at the Ege University Faculty of Dentistry Research Laboratory. Acrylic-embedded specimens were positioned with the enamel surface parallel to the indenter. Prior to measurements, the device was calibrated using a standard reference block according to the manufacturer’s instructions to ensure measurement accuracy and reproducibility. A load of 200 g was applied for 15 s at three separate points on each enamel surface. Vickers hardness numbers (VHN) were automatically calculated by the device software, and the mean of the three measurements was recorded as the baseline surface microhardness value.

### 2.4. pH Cycling Protocol

Specimens were subjected to a pH cycling regimen consisting of alternating demineralization and remineralization phases. Initially, samples were immersed in artificial saliva (pH 6.8) for 16 h. Subsequently, each specimen was transferred to 90 mL of demineralization solution for 8 h [18].

The demineralization solution contained 2.2 mM calcium nitrate [Ca(NO_3_)_2_], 2.2 mM monopotassium phosphate [KH_2_PO_4_], and 0.1 ppm sodium fluoride [NaF], with the pH adjusted to 4.5 using 50 mM acetic acid [C_2_H_4_O_2_]. One complete cycle consisted of 16 h in artificial saliva followed by 8 h in the demineralization solution, totaling 24 h per cycle. The cycling procedure was repeated for 30 days, with solutions renewed twice daily. Throughout the experiment, specimens were maintained in a water bath at 37 °C under low-speed agitation (50 rpm). Artificial saliva was prepared according to ISO/TR 10271 [18,19].

### 2.5. Surface Microhardness Measurement After Demineralization

Following the pH cycling protocol, surface microhardness measurements were repeated using the same parameters as the baseline assessment. The mean of three indentations per specimen was recorded as the post-demineralization surface microhardness value.

### 2.6. Application of Microabrasion Technique (Groups G1 and G3)

Microabrasion was performed exclusively in Groups G1 and G3 as a surface pretreatment prior to resin infiltration. A commercially available microabrasion paste containing 6.6% hydrochloric acid (Biowhiten, Istanbul, Turkey) was applied using a rubber cup mounted on a low-speed handpiece operating at approximately 1000 rpm. The paste was actively rubbed onto the enamel surface under light pressure for 10 s per application. This procedure was repeated three times, resulting in a total application time of 30 s per specimen. Between applications, the enamel surfaces were rinsed with water for 10 s and gently air-dried [20].

All microabrasion procedures were performed by a single calibrated operator to ensure standardization and minimize operator-related variability. The combined use of microabrasion and hydrochloric acid etching was intentionally selected to create a worst-case surface conditioning model, maximizing enamel surface permeability under controlled in vitro conditions and allowing comparability with previous experimental studies employing aggressive pretreatment protocols [20,21,22]. Following microabrasion, standard ICON etching with 15% hydrochloric acid was applied as part of the resin infiltration protocol.

### 2.7. Application of the ICON Resin Infiltrant (Groups G3 and G4)

Following microabrasion in Group G3 and directly after lesion formation in Group G4, resin infiltration was performed using ICON (DMG, Hamburg, Germany) according to the manufacturer’s instructions. Briefly, the lesion area was etched with ICON-Etch for 2 min, then rinsed with water for 30 s and dried with oil- and water-free air. The lesion was subsequently dehydrated with ICON-Dry (ethanol) for 30 s and air-dried thoroughly. ICON-Infiltrant was then applied and allowed to infiltrate for 3 min; excess material was removed and the surface was light-cured for 40 s. A second layer of ICON-Infiltrant was applied for 1 min, excess was removed, and the material was light-cured again for 40 s. During infiltration, the material was protected from direct operating light to prevent premature setting.

### 2.8. Application of the Experimental Resin Infiltrant (Groups G1 and G2)

The experimental resin infiltrant was applied using the same standardized application protocol as the ICON resin. Briefly, 15% hydrochloric acid was applied to the enamel surface for 2 min, followed by thorough rinsing and air-drying for 30 s. Subsequently, 99% ethanol was applied for 30 s and air-dried. A TEGDMA-based resin matrix (TEGDMA 80–95 wt%, camphorquinone < 2 wt%) (Table 1) was applied twice: the first application for 3 min followed by light curing for 40 s, and the second application for 1 min followed by light curing for 40 s. After infiltration, the enamel surfaces were polished.

### 2.9. Examination of Resin Penetration Depth

Penetration depth measurements were conducted on a separate subset of specimens (*n* = 6 per group), as the sectioning, staining, bleaching, and confocal laser scanning microscopy (CLSM) procedures required for this analysis are inherently destructive and preclude repeated testing. Therefore, these specimens were not included in the surface microhardness evaluations.

After resin infiltration, specimens were sectioned along the buccolingual plane using a water-cooled diamond saw. Resin penetration was assessed using a dual-fluorescence staining technique combined with confocal laser scanning microscopy (CLSM), as described by Paris et al. [21] Infiltrated regions were identified by red fluorescence, while non-infiltrated porous areas appeared green. Penetration depth was measured at three standardized locations per section using image analysis software, and mean values were calculated for statistical analysis.

### 2.10. Statistical Analysis

Statistical analyses were performed after confirming data normality (Shapiro–Wilk test) and homogeneity of variances (Levene’s test). Baseline microhardness values were compared using one-way ANOVA to verify intergroup homogeneity prior to demineralization. Microhardness (VHN) values obtained at three time points (baseline, post-demineralization, and post-treatment) were analyzed using a repeated-measures mixed-effects model to assess the effects of time, group, and their interaction. When significant differences were detected, pairwise comparisons were conducted using Tukey’s post hoc test. Penetration depth data were analyzed using one-way ANOVA, followed by Tukey’s post hoc test when appropriate. All results were expressed as mean ± standard deviation (SD), with statistical significance set at *p* < 0.05.

This study has certain methodological limitations inherent to its in vitro design. The experimental conditions cannot fully replicate the complex biological, chemical, and mechanical factors present in the oral environment. In addition, surface microhardness measurements primarily reflect superficial changes, while penetration depth analysis required destructive sample preparation, preventing repeated measurements on the same specimens.

## 3. Results

### 3.1. Microhardness Analysis

The microhardness values of the study groups at baseline, after demineralization, and after resin infiltration are presented in Table 2. Baseline microhardness values were comparable among all groups, confirming initial intergroup homogeneity. Following the demineralization protocol, a pronounced reduction in enamel surface microhardness was observed in all groups, indicating successful induction of artificial enamel lesions. This reduction was statistically significant, as demonstrated by the mixed-effects model, which revealed a significant main effect of time (*p* = 0.045).

After resin infiltration, a partial recovery in surface microhardness was observed across all groups compared with the demineralized state. However, none of the experimental groups, with the exception of Group 2, reached baseline microhardness values, indicating incomplete mechanical recovery. No statistically significant differences were detected between the groups at any measurement time point, and the interaction effect between time and group (time × group) was also not significant, suggesting a similar microhardness response pattern for all resin systems and pretreatment protocols.

**Figure 1:**
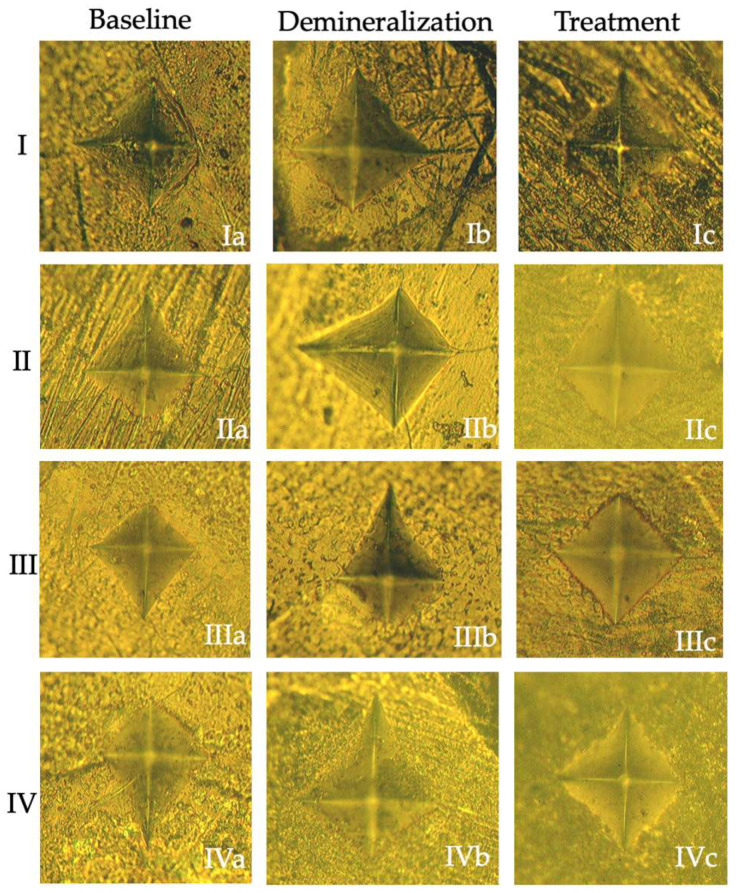
Representative microhardness indentation images of the groups. Baseline (**Ia**–**IVa**), demineralization (**Ib**–**IVb**), and treatment (**Ic**–**IVc**) conditions are shown for Groups I–IV.

**Figure 2:**
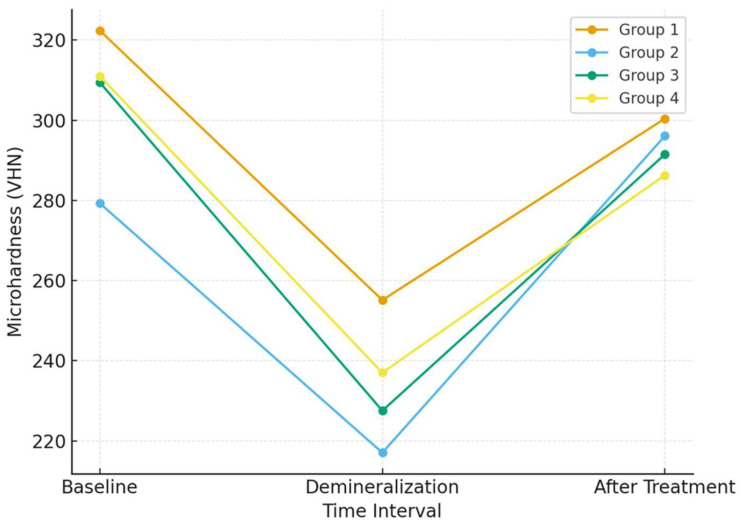
Changes in enamel surface microhardness (VHN) at baseline, after demineralization, and after resin infiltration for all study groups. Values are presented as mean ± standard deviation (SD). No statistically significant differences were observed between groups at corresponding time points (*p* > 0.05).

The overall trend of microhardness reduction after demineralization and subsequent partial recovery following resin infiltration is illustrated in Figure 1 and Figure 2.

### 3.2. Penetration Depth Analysis

Penetration depth analysis revealed statistically significant differences among the four experimental groups (*p* < 0.05). Specimens treated with the ICON resin infiltrant (Groups 3 and 4) demonstrated significantly greater penetration depths compared with those treated with the experimental resin formulation.

Among all groups, Group 3 (ICON with microabrasion) exhibited the greatest penetration depth (412.90 ± 18.44 µm), followed by Group 4 (ICON without microabrasion; 356.76 ± 6.80 µm). In contrast, the experimental resin groups showed markedly lower penetration depths, with mean values of 278.50 ± 18.06 µm for Group 1 and 239.30 ± 14.10 µm for Group 2 (Table 3).

When groups subjected to microabrasion (Groups 1 and 3) were compared with their respective non-microabraded counterparts (Groups 2 and 4), microabrasion was associated with a significant increase in penetration depth for both resin systems. Group 1 demonstrated significantly deeper penetration than Group 2, and similarly, Group 3 exhibited significantly greater penetration depth compared with Group 4 (*p* < 0.05). These findings indicate that microabrasion enhanced enamel surface permeability, thereby facilitating deeper monomer infiltration.

**Figure 3:**
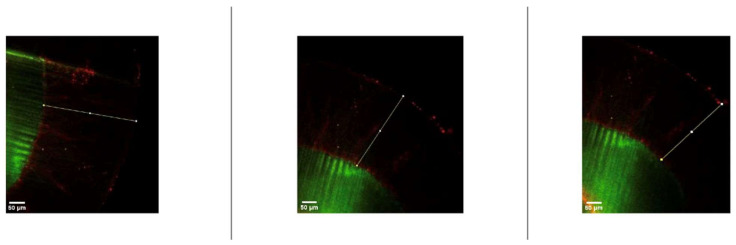
Representative confocal laser scanning microscopy (CLSM) images showing resin penetration depth in experimental resin infiltration groups. Red fluorescence indicates infiltrated regions, whereas green fluorescence represents non-infiltrated porous enamel areas. White lines show the penetration depth of the resin infiltration.

**Figure 4:**
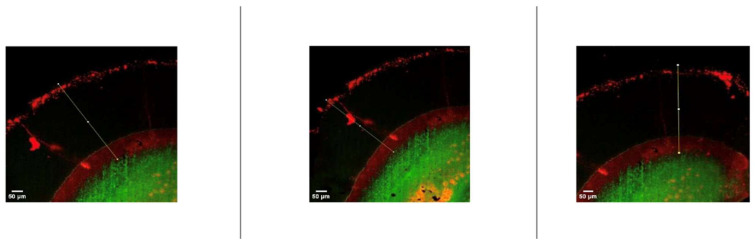
Representative confocal laser scanning microscopy (CLSM) images showing resin penetration depth in ICON resin infiltration groups. Red fluorescence indicates infiltrated regions, whereas green fluorescence represents non-infiltrated porous enamel areas. White lines show the penetration depth of the resin infiltration.

Representative confocal laser scanning microscopy images illustrating resin penetration depths assessed using the dual-fluorescence staining technique are presented in Figure 3 and Figure 4.

## 4. Discussion

Resin infiltration is widely recognized as a minimally invasive and tissue-preserving approach for the management of non-cavitated enamel lesions, particularly white spot lesions (WSLs) [23]. The primary objective of this technique is to arrest lesion progression by occluding the porous enamel structure and limiting further acid diffusion within the lesion body. However, it is critical to emphasize that resin infiltration should not be interpreted as a true remineralization strategy. In the present study, although partial recovery of enamel surface microhardness was observed following resin infiltration, none of the tested materials were able to restore microhardness values to baseline levels. This finding clearly indicates that the observed improvement reflects surface stabilization rather than biological or mineral-based enamel regeneration.

Resin infiltration exerts its protective effect predominantly through mechanical sealing of enamel microporosities rather than through intrinsic reinforcement of the mineral structure. Consequently, increases in surface microhardness should be interpreted cautiously and regarded as an indirect mechanical effect resulting from resin occupation of the porous lesion network. The absence of complete microhardness recovery across all groups further supports the concept that resin infiltration provides lesion stabilization rather than functional remineralization of enamel tissue. It should be acknowledged that surface microhardness measurements primarily reflect superficial mechanical changes and may not fully capture subsurface mineral alterations or the overall mechanical integrity of infiltrated enamel; therefore, complementary approaches such as cross-sectional hardness profiling or nanoindentation could provide more comprehensive insight in future investigations [12,21,22].

The inclusion of microabrasion as a surface pretreatment in the present study was deliberately chosen despite its well-documented limitations, including irreversible enamel loss, in order to establish a controlled worst-case surface conditioning model. This approach was not intended to simulate routine clinical practice but rather to maximize enamel surface permeability and to investigate the upper limits of resin infiltration under standardized in vitro conditions. By applying an intensified pretreatment protocol, the study aimed to isolate and evaluate the true penetration capacity of the infiltrant materials independent of superficial diffusion barriers, thereby enabling a more sensitive comparison between the experimental resin formulation and the commercially available ICON system. Such an approach also ensured methodological comparability with previous in vitro studies employing aggressive surface conditioning to assess maximal infiltration potential. Consequently, the observed effects of microabrasion should be interpreted as a methodological tool for performance assessment rather than as a recommendation for routine clinical application [24,25,26].

Surface conditioning plays a pivotal role in the effectiveness of resin infiltration, as the hypermineralized surface layer of early enamel lesions represents a diffusion barrier that limits monomer penetration into the lesion body. In the present study, 15% hydrochloric acid was used for surface conditioning prior to infiltration, in line with previous investigations demonstrating its superior ability to remove the superficial enamel layer compared with phosphoric acid. Meyer-Lueckel et al. reported that 15% hydrochloric acid can remove approximately 40 μm of the hypermineralized surface layer, thereby facilitating access to the subsurface lesion zone [22,27].

Microabrasion was evaluated as an adjunctive surface pretreatment to further enhance resin penetration. The results demonstrated that microabrasion significantly increased penetration depth in both resin systems tested. However, this effect must be interpreted with caution, as hydrochloric acid-based microabrasion systems are well documented to induce irreversible enamel loss through surface subtraction rather than structural reinforcement. Accordingly, the increased penetration depth observed following microabrasion may primarily reflect enhanced surface removal rather than a true improvement in infiltration efficiency within an intact enamel substrate. Importantly, greater resin penetration depth should not be interpreted as an inherent indicator of superior clinical performance or enamel reinforcement per se, particularly in the absence of corresponding improvements in surface mechanical properties.

Importantly, despite the significant increase in penetration depth, microabrasion did not result in a corresponding improvement in enamel surface microhardness compared with non-microabraded groups. This dissociation suggests that deeper resin penetration does not necessarily translate into superior mechanical reinforcement of the enamel surface. Therefore, penetration depth alone should not be regarded as a definitive indicator of enhanced clinical performance [28,29].

The findings of the present study are in agreement with previous investigations reporting that resin infiltration results in lesion stabilization rather than complete remineralization, as evidenced by partial recovery of surface microhardness without restoration of baseline values. Consistent with earlier studies, the commercially available ICON system demonstrated greater penetration depth compared with experimental or non-optimized resin formulations, which has been attributed to its lower viscosity and higher penetration coefficient. Moreover, the enhancing effect of aggressive surface conditioning on resin penetration observed in this study has also been reported by other authors, although such protocols are generally regarded as experimental and not routinely recommended for clinical practice.

An additional consideration relates to the cumulative effect of the dual acidic pretreatment protocol used in this study. The sequential application of hydrochloric acid-based microabrasion followed by 15% hydrochloric acid etching may result in additive enamel surface loss. While such an approach may be justified under controlled in vitro conditions to investigate maximum infiltration potential, its routine clinical application remains questionable, particularly in teeth with limited remaining enamel thickness [20].

From a clinical perspective, excessive surface conditioning may predispose enamel to over-etching and surface weakening, potentially offsetting the benefits of enhanced resin penetration. Alternative strategies, such as prolonged etching without prior microabrasion or modification of acid concentration and application time, may achieve sufficient surface permeability while minimizing irreversible tissue loss. Previous studies have suggested that extended etching alone can improve resin penetration without the additional mechanical abrasion associated with microabrasion [30,31].

Penetration depth analysis further demonstrated a clear superiority of the ICON resin infiltrant compared with the experimental formulation. The lower penetration depth observed for the experimental resin compared with ICON may be attributed to material-related differences such as viscosity and wettability, which directly influence monomer diffusion into narrow enamel microporosities. ICON is specifically optimized for infiltration and exhibits a higher penetration coefficient, enabling deeper resin diffusion within the lesion body [29,32]. Despite this difference in penetration depth, no significant differences in surface microhardness recovery were observed between materials, indicating that deeper subsurface infiltration does not necessarily translate into improved surface mechanical properties. This finding supports the concept that surface microhardness primarily reflects superficial stabilization rather than the extent of subsurface resin penetration.

Overall, the findings of the present study indicate that while microabrasion can enhance resin penetration depth under experimental conditions, its clinical use should be selective rather than routine. The potential benefits of increased penetration must be carefully balanced against the risk of irreversible enamel loss, and deeper infiltration should not be automatically equated with superior clinical outcomes. Resin infiltration should therefore be regarded as a microinvasive lesion-arrest strategy, with surface pretreatment protocols tailored to lesion characteristics and enamel preservation principles.

## 5. Conclusions

Within the limitations of this in vitro study, the following conclusions can be drawn:

- Resin infiltration resulted in partial recovery of enamel surface microhardness after artificial demineralization; however, baseline hardness values were not fully restored, indicating surface stabilization rather than true remineralization.
- No significant differences in surface microhardness recovery were observed between the experimental resin infiltrant and the ICON system.
- The experimental resin infiltrant demonstrated measurable penetration into demineralized enamel lesions but exhibited significantly lower penetration depth compared with the commercially available ICON system.
- Microabrasion significantly increased resin penetration depth for both infiltrant materials; however, this effect did not translate into improved surface microhardness and should be interpreted cautiously due to the risk of irreversible enamel loss.
- These findings support the study objectives and hypotheses by confirming that the experimental resin can infiltrate demineralized enamel and contribute to lesion stabilization, while highlighting that its infiltration efficiency remains inferior to that of the ICON system.
- Overall, resin infiltration should be regarded as a lesion-arrest strategy, and further optimization of experimental resin formulations is required to achieve penetration performance comparable to established commercial systems.

## Figures and Tables

**Table 1:** Description of the composition of resin infiltrants.

Resin Infiltrants	Composition
Experimental Resin Infiltrant	TEGDMA 80–95 wt%, camphorquinone < 2 wt%
ICON Resin Infiltrant	TEGDMA 70–95 wt%, camphorquinone < 2.5 wt%

**Table 2:** Enamel surface microhardness values (VHN) at baseline, after demineralization, and after resin infiltration for all study groups.

Groups	Pretreatment Protocol	Baseline (Mean ± SD)	Demineralization (Mean ± SD)	Treatment (Mean ± SD)
Group 1	With microabrasion	322.38 ± 21.26 ^a^	255.12 ± 16.47 ^b^	300.40 ± 40.91 ^a^
Group 2	Without microabrasion	279.28 ± 40.17 ^a^	217.04 ± 19.39 ^b^	296.08 ± 42.74 ^a^
Group 3	With microabrasion	309.44 ± 32.26 ^a^	227.52 ± 48.39 ^b^	291.48 ± 46.32 ^a^
Group 4	Without microabrasion	311.00 ± 39.46 ^a^	237.00 ± 34.45 ^b^	286.28 ± 29.00 ^a^

Values are presented as mean ± standard deviation (SD). Different lowercase superscript letters within the same column indicate statistically significant differences (*p* < 0.05).

**Table 3:** Resin penetration depth (µm) values for all study groups following resin infiltration.

Groups	Pretreatment Protocol	Resin Type	*n*	Penetration Depth (Mean ± SD, µm)
Group 1	With microabrasion	Experimental resin	6	278.5 ± 18.06 ^a^
Group 2	Without microabrasion	Experimental resin	6	239.30 ± 14.1 ^b^
Group 3	With microabrasion	ICON resin	6	412.9 ± 18.44 ^c^
Group 4	Without microabrasion	ICON resin	6	356.76 ± 6.08 ^d^

Values are presented as mean ± standard deviation (SD). Different lowercase superscript letters indicate statistically significant differences between groups (*p* < 0.05).

## Data Availability

The original contributions presented in the study are included in the article, further inquiries can be directed to the corresponding author.
